# Knitted Strain Sensors: Impact of Design Parameters on Sensing Properties

**DOI:** 10.3390/s140304712

**Published:** 2014-03-07

**Authors:** Ozgur Atalay, William Richard Kennon

**Affiliations:** School of Materials, University of Manchester, Manchester, M60 1QD, UK; E-Mail: Richard.kennon@manchester.ac.uk

**Keywords:** conductive yarn input tension, elastomeric yarn, intelligent textiles, knitted sensor, strain sensor, production parameters, silver-plated nylon yarn

## Abstract

This paper presents a study of the sensing properties exhibited by textile-based knitted strain sensors. Knitted sensors were manufactured using flat-bed knitting technology, and electro-mechanical tests were subsequently performed on the specimens using a tensile testing machine to apply strain whilst the sensor was incorporated into a Wheatstone bridge arrangement to allow electrical monitoring. The sensing fabrics were manufactured from silver-plated nylon and elastomeric yarns. The component yarns offered similar diameters, bending characteristics and surface friction, but their production parameters differed in respect of the required yarn input tension, the number of conductive courses in the sensing structure and the elastomeric yarn extension characteristics. Experimental results showed that these manufacturing controls significantly affected the sensing properties of the knitted structures such that the gauge factor values, the working range and the linearity of the sensors varied according to the knitted structure. These results confirm that production parameters play a fundamental role in determining the physical behavior and the sensing properties of knitted sensors. It is thus possible to manipulate the sensing properties of knitted sensors and the sensor response may be engineered by varying the production parameters applied to specific designs.

## 1. Introduction

Intelligent textiles or electro-textiles may be defined as flexible textile structures that have the capability to react to environmental stimuli [1]. The sensing capability of electro-textiles can be utilised to measure some physiological parameters of the human body, such as respiration rate [2,3], body temperature [4,5], heart rate and [6,7] body posture [8,9]. There is a plethora of production methods and raw materials which may be employed in the manufacture of electro-textile platforms. However, one of the main issues which determine the effectiveness of the resulting structure is the selection of an appropriate production method and the incorporation of suitable raw materials to meet such requirements as working range, responsivity, repeatability and the response time required for the target application. The raw materials must also be compatible with the intended textile production machinery and the required textile properties of the finished product. Traditional textile production methods such as knitting [10–13], weaving [14–18] and embroidery [19–21] can be used to create the basis of sensing structures. Different kinds of coating materials and conductive yarns such as metal yarns, metal coated yarns or spun yarns incorporating metal fibres which can be used as raw materials to create conductive elements. These materials act as sensing components or transmission lines within the finished structure [22–25].

One of the growing research areas of electro-textiles is the creation of strain sensing structures. The working principle of textile-based strain sensors originally relied on the traditional metal-based strain gauges. However, textile-based sensors introduce a higher level of elasticity into the material due to the inherent properties of textile structures. Piezoresistive sensor technology is currently common method of creating textile-based strain sensors along with capacitive, inductive, and impedance sensors. This is because piezoresistive technology offers advantages over the other sensor technologies; “high responsivity/small size/simplicity/repeatability” [26]. Capacitive sensors in electro-textiles area are mainly created for pressure detection [27,28], and construction of these sensors is more complex due to the usage of dielectric material and the requirement for consistent separation of conductive panels at all times. They are also more sensitive to changes in temperature or humidity [29]. In spite of the fact that inductive or impedance sensors demonstrate good results for the measurement of physiological parameters of the human body; signal interference has proven to be a serious problem during trials [30].

The operation of the piezoresistive strain sensor is based on the variation of electrical charge concentration in response to externally applied strain [31], which modifies the shape of the piezoresistive element. Piezoresistive textile strain sensing structures may be created either at the yarn level or at the fabric level and desirable sensing properties for proposed applications can be obtained by varying the conductive materials or the textile structures. However, elasticity is an essential property of conductive yarns, particularly if they are specifically intended for use as strain sensors. To realise this aim, thermoplastic or rubber elastomers with carbon black particles are commonly used to create sensing structures [32,33]. In another study, flexible silicon skins were stitched onto textile fabric for enhanced flexibility of the sensing region [34].

An alternative approach for the creation of textile sensors is to employ elastomeric yarn as a core material and conductive yarn as a helical winding around the extensible core [35,36]. This multi-component approach is necessary because conductive yarns such as metal filament yarns, yarns incorporating metal staple fibres into the spinning process or coated yarns do not on their own offer sufficient elasticity for the creation of strain sensors at the yarn level.

The creation of sensing structures at the fabric level is the most common method that is employed and knitting technology is widely preferred as it offers a relatively high level of elasticity which is inherent in the looped structure. At this level, conductive yarns offering moderate levels of stretch and recovery are sufficient to create sensing structures as the knitted structure itself provides sufficient operational elasticity [37]. The elasticity level of fabric can also be improved by knitting conductive yarns along with elastomeric yarns. At this stage, the design of the sensing structure is a crucial part of the work, as it directly affects the sensing mechanism of the eventual device. Knitted sensing structures can be created by using only conductive yarns in the creation of the reactive panel. Such devices rely on the natural structural elasticity of the knitted fabric to provide recovery after stress deformation. However, this method has some drawbacks, including the limited elasticity of the sensing panel, the small working range of the sensor, and the necessary deformation of conductive yarns during repetitive usage; these factors affect the responsivity and the reliability of the sensor in real time applications. Thus, conductive yarns are commonly knitted along with non-conductive yarns and this improves the dimensional stability of the detector and it offers the advantage that multiple sensing areas may be created within a single knitted structure by selective introduction of the conductive yarn [1][11]. This method opens alternative design possibilities, one of which is the creation of consecutive conductive courses within a non-conductive base structure [38]. The sensing mechanism for all these approaches relies predominantly on the electrical resistance inherent in the variable contact between overlapping yarns within the knitted composition [39]. However, during extended usage, degradation of the conductive parts is inevitable due to repetitive frictional action between the overlapping yarns. Thus, a new modified approach has been adopted to overcome these problems. In this design, a pre-tensioned knitted sensor has been created by embedding conductive yarns into an interlock-knitted base structure in a series of single loops. The conductive yarn itself is affected only minimally by the applied forces as the sensor is extended and released and hence it retains its structural properties over a longer period of operation [10].

In the current literature, most of the work on textile sensors is focused on the electro-mechanical properties of these devices. Since electro-textiles represent a relatively new area of investigation, this work is concerned predominantly with the production of working prototypes or pre-prototypes; there is little investigation of the textile properties or the production parameters of the sensing mechanisms.

In this paper, the effect of integrating elastomeric yarns with different linear yarn densities into sensing mechanisms is investigated for specific designs of knitted sensor. The effect on the sensing mechanism of varying the input tension of the conductive yarn is also explored. In addition, the number of courses of conductive yarn used in the sensors has been varied. Thus, the effect on the sensing properties of an increased or decreased number of course of conducting yarn can be considered. The following section describes the electro-mechanical theory and production of knitted strain sensors, followed by the testing methods used to assess the electromechanical properties of the sensors. The third part of this work reports the results obtained from the experimental procedures and promotes a discussion of the electro-mechanical properties of the sensors.

## 2. Materials and Methods

### 2.1. Production of Knitted Strain-Sensing Fabrics

Initially, a single design of knitted strain-sensing fabric was devised, comprising a silver coated nylon conductive yarn with 2 Ω/cm linear resistance and insulating core-spun Lycra yarn. Conductive yarn was purchased from the Swicofil AG (Emmenbruecke,Switzerland) Three different variations of the basic knitted sensing fabric were created using 800 dtex (the mass in grams of 10,000 metres of yarn), 570 dtex and 156 dtex gauge Lycra elastomeric yarns respectively. The elastomeric core of each Lycra yarn was wrapped with a double covering of continuous filament nylon. The three variants of the sensing structure were manufactured on a Shima Seiki SES 122-S ten gauge computerised flat-bed knitting machine. Table 1 shows the production parameters of each type.

**Figure 1.**
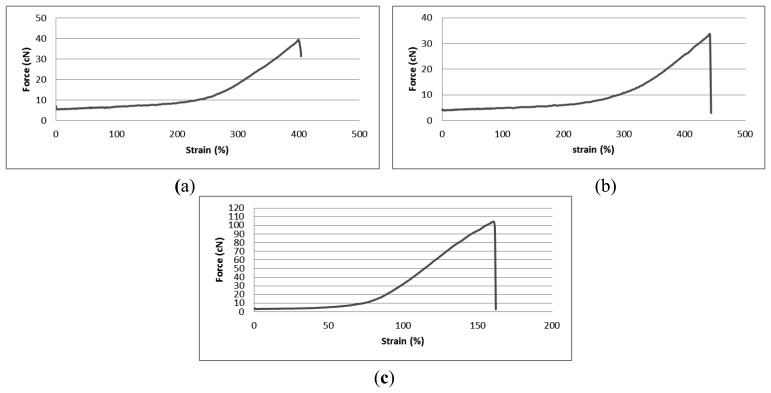
Force-strain graphs of three different gauges of elastomeric yarn. (**a**) Knitted structure with 800 dtex elastomeric yarn. (**b**) Knitted structure with 570 dtex elastomeric yarn. (**c**) Knitted structure with 156 dtex elastomeric yarn.

Initially, the tensile properties of the three elastomeric yarns were determined. Whilst the 800 dtex and 570 dtex core-spun Lycra yarns with a 1/33/10 PA 6.6 covering were supplied by the same company, the 156 dtex with 44/33/2 PA6.6 with a 78/46/2 PA 6.6 covering was obtained from a different source. Figure 1 shows force-strain graphs of the three elastomeric yarns up to the breaking point.

The run-in tension of the elastomeric yarns was maintained at 8 cN for all three gauges of wrapped Lycra. Thus, the strain value of 8 cN during tensile testing will provide information about the behavior of elastomeric yarn during knitting; it will reveal the fabric tightness during the manufacturing stage. The higher the yarn extension at the 8 cN run-in force for the Lycra yarns, the tighter fabric is. The Lycra stitches will be shorter than the nominal stitch length with the difference depending on the degree of elastic extension of the elastomeric yarn during the knitting process because after the formation of the stitches, when the fabric is removed from the physical constraints of the knitting machine, the yarn tension will reduce and the knitted structure will relax. Table 2 shows the elongation values of elastomeric yarns at the 8 cN applied input yarn tension.

**Figure 2.**
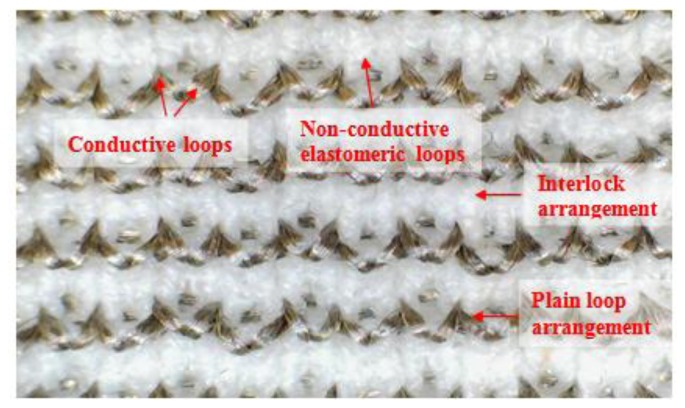
Technical face of sensor design includes conductive & elastomeric yarns.

Two yarn feeders were used to fabricate samples. The first feeder was responsible for creating an interlock structure using elastomeric yarn and the second feeder was used for embedding conductive yarn into this structure in a plain knit arrangement. The conductive yarn was used to create conductive loops and they were positioned only on the technical face of the fabric. The reason for this arrangement was to avoid contact between the conductive yarns and the human body; it is an important safety concern to avoid contacting the skin with the conductive parts of wearable sensors. A secondary consideration is that the conductivity of the human skin would affect the signal. Thus, the three types of strain sensing fabric were manufactured using an interlock arrangement and the conductive yarn was embedded into this interlock structure in a series of single loops. The technical face of a knitted sample is shown in Figure 2.

Each type of sample was developed with a sensing area of 36 conductive wales and six conductive courses. However, conductive yarn was not knitted into every course; it was an inherent part of the design that non-conductive courses would be knitted to maintain physical separation between parallel lines of conductive yarn.

Smooth withdrawal and constant run-in tension in the elastomeric yarn was an important concern to ensure manufacturing repeatability of the fabric structures. In order to achieve this aim, a “BTSR” constant tension feeder was used and the run-in yarn tension was kept at 8 cN for each type of elastomeric yarn. The feed control device was located on the side of the flat-bed knitting machine and enabled feeding of yarn at the programmed tension to the knitting zone for the duration of the manufacturing process. By controlling the yarn tension accurately, uniform stich length can be generated throughout the fabric structure.

The 800 dtex elastomeric yarn sample with an 8cN input tension was selected for further experimentation. The conductive yarn input tension had been regulated by using a second “BTSR” constant tension feeder. Hence the conductive yarn input tension has been applied at 5 cN, 10 cN and 20 cN in order to monitor the effect of conductive yarn input tension on the sensing mechanism. Finally, samples with a different number of conductive courses were produced with 800 dtex elastomeric yarn at 8 cN elastomeric yarn input tension. The number of conductive courses was varied and examples with four and six courses of silver-plated nylon yarn were incorporated into the sensor design in order to see the effect of this parameter on the responsivity of the device.

### 2.2. Electromechanical Theory and Structural Design

**Figure 3.**
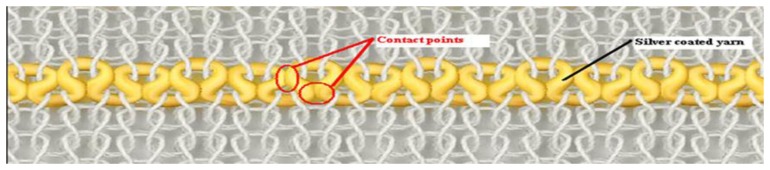
Schematic diagram of textile sensor showing the conductive loops in a single knitted course and the contact points between successive conducting loops.

The detection mechanism of the knitted sensor is based on the specific design of the conductive yarn in the fabric structure that enables the sensor to change its electrical resistance with in response to variations in strain. There are two predominant factors which are responsible for change of electrical resistance in response to strain; the resistance changes due to extension of the conductive yarn within the structure, and the contact points between successive knitted loops of conducting yarn are pulled apart and cause the sensor to change its resistance. However, the degree of influence of these factors varies with fabric structure, the type of conductive yarn and the applied strain level. If the applied strain is not high enough to elongate the conductive yarn within the knitted structure, relocation of the contact areas between adjacent loops of conducting yarn becomes the main controlling factor for the behaviour of the sensing mechanism. To illustrate the situation, Figure 3 shows a schematic diagram of one conductive course within the structure.

As shown in Figure 3, due to the usage of elastomeric yarn in the fabric structure, conductive yarn loops make contact with adjacent loops at their heads and limbs also at their sinker loops which are pressed together and according to Holm's contact theory [40]:

(1) $$R_{C}=\frac{\rho }{2}\sqrt{\frac{\pi H}{nP}}$$

where:

*R_C_*= contact resistance;
ρ = electrical resistivity;
H = material hardness;
n = number of contact points;
p = contact pressure.

From Equation (1), it is seen that the material hardness and electrical resistivity are constant for a given conductive yarn but the number of contact areas and the contact pressure change depends on the applied strain Thus, higher contact pressure and an increased number of contact areas between the conductive parts reduce the contact resistance. In this specific sensor design, the contact pressure and the number of contact areas between the conductive loops demonstrate their highest values prior to extension of the knitted sensor but during the force loading stage. This is because the application of a uniaxial tensile force reduces the strength of contact between the conductive loops. Thus, the contact pressure, the contact area and the number of contacting points between conductive loops lessens depending on the level of applied strain. Hence the overall electrical resistance of the sensor increases with strain due to the increase of contact resistance.

The gauge factor (GF) is an important parameter for strain sensors and it determines the sensitivity of the sensor. The GF can be calculated as follows:

(2) $$GF=\frac{\frac{\Delta R}{R}}{ɛ}$$

where:

ΔR = the change in the resistance;
R = the initial resistance (the resistance before extension);
ε = the strain value.

### 2.3. Test Procedure for Knitted Strain Sensors

**Figure 4.**
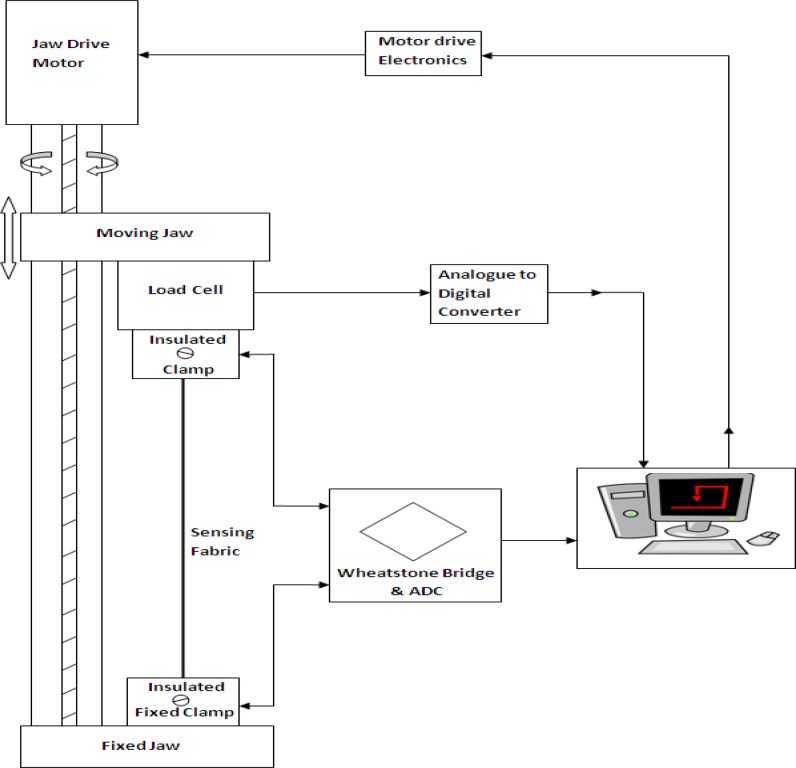
Schematic diagram of the tensile and resistance measuring equipment.

Initially, sensing structures were subjected to levels of up to 40% strain in the course direction in order to determine their response characteristics. The extension level of 40% was chosen to reflect typical human body extensions, as the proposed sensors may be used for monitoring human body movements. Electro-mechanical measurements of the various samples were performed under multi-cyclic tensile stress using a Zwick/Roell BTC-FR2.5TS.D09 tensile testing machine to apply repeated mechanical extension and deformation. The change of resistance was measured concurrently with the applied strain by using the tensile testing equipment in combination with a Wheatstone bridge analytical arrangement, as shown in Figure 4.

Experimental data were recorded using the TestExpert software. The tensile testing machine has a fixed jaw on its base-plate and a moveable crosshead which may be driven at a range of pre-determined speeds. In this research, samples were tested with a constant rate of extension of 120 mm/min with a full test comprising 20 repeated cycles.

## 3. Results and Discussion

### 3.1. Effect of Elastomeric Yarn Type on Sensing Properties

**Figure 5.**
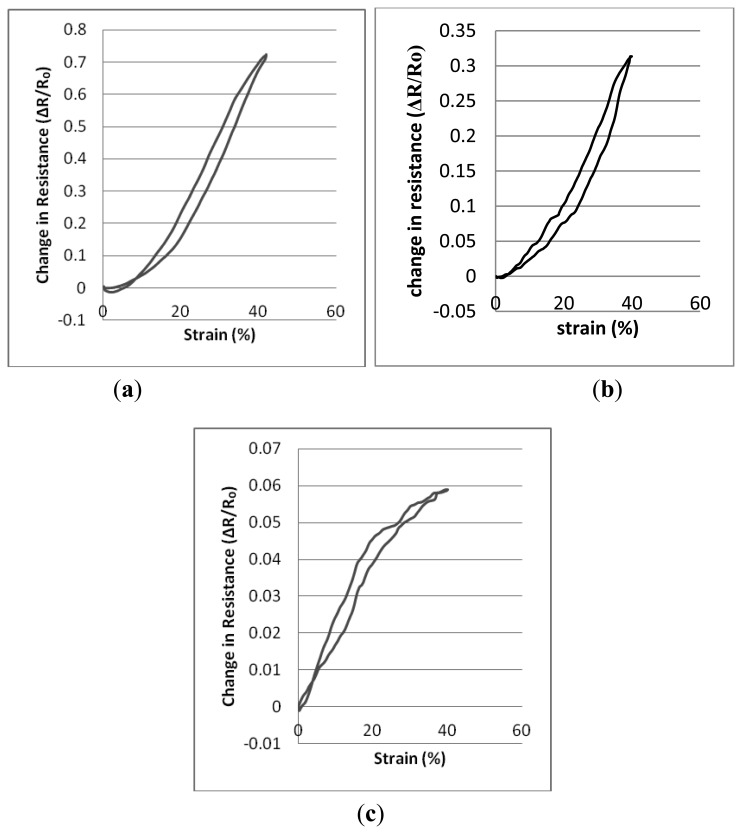
Relative change in resistance-strain graphs of three types of sensors. (**a**) Type 1; (**b**) Type 2; (**c**) Type 3.

Tables 3, 4, and 5 present descriptions of the resistance-strain curves in terms of the statistical parameters. A full sequence of 20 cyclic repeats was used for each of the calculations. The sensing fabrics were tested between 0% and 40% strain levels at an extension speed of 120 mm/min and there were no dwell times at either the lowest or the highest strain levels. The sensors' working range, the R**^2^** values which describe the quality of the fitted line and the gauge factor values were calculated individually for each repeat. Thereafter, statistical analysis has been performed based on these repeats. Figure 5 shows the relative change in resistance against strain for the three types of sample whilst they are being subjected to cyclic tensile testing. Graphs were plotted by averaging the 20 cyclic measurements.

It may be seen in the graphics of Figure 5 that there are actually two hysteresis loops described by the curves. Thus, the working range of the knitted sensors can be taken into account as being between the finishing strain values of the first hysteresis loops and the 40% strain values. Also, the maximum hysteresis values of sensors are 3.45%, 4% and 5.20% for Type 1, Type 2 and Type 3 sensors respectively. As may be seen from the various graphs and tables, the three types of sensor show differing behavior and different gauge factors in response to applied strain. Firstly, while Type 1 and Type 2 sensors can be characterised by one linear region over their entire working range. Linear working ranges are between 8.405%–40% and 2.624%–40% for Type 1 and Type 2 sensors respectively. Type 3 sensors can be characterised by the fact that they demonstrate two separate regions. The reason for this behavior is that; Type 1 and Type 2 sensors are structurally more compact than the design parameters used for Type 3, Type 1 and Type 2 sensors have higher stitch densities in compared to Type 3 sensors due to intrinsic property of elastomeric yarns. Thus, higher strain rates are needed to separate the contact points of the loops of conducting yarn from each other in the more compact knitted structures. In addition, Type 3 sensors exhibit the lowest gauge factor values compared to the other two types because Type 3 sensors are less compact structures and show reduced contact area and lower contact pressure between the conductive points of adjacent knitted loops and this has a significant effect on the gauge factor. Hence, Type 3 sensors have fewer conductive touching points and an applied strain does not contribute such a significant change in resistance as for Type 1 and Type 2 sensors. Although Type 2 sensors are the most compact of the three differing sensing fabrics, their gauge factor values are not as not high as those of Type 1 sensors due to the reduced conductive contact points compared with the Type 1 samples which have been produced using thicker (and therefore stronger) elastomeric yarn.

### 3.2. Effect of Conductive Yarn Input Tension on Sensing Properties

To investigate the effect of variations in the knitting tension during the manufacture of knitted sensors, the input tension of the conductive yarn was set at 5 cN, 10 cN and 20 cN in order to produce 3 different variations on the basic form of the knitted sensors. Prior to analysing the resistance-strain data, the resistance of the samples was measured. Table 6 shows the resistance values of the three knitted sensors at zero strain.

**Figure 6.**
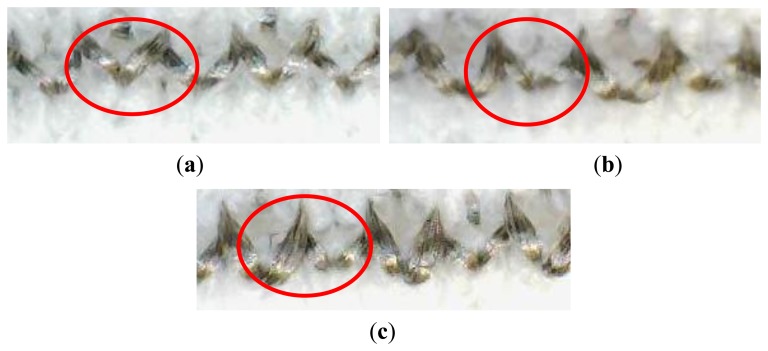
Magnified images of conductive loops in part of a single knitted course (**a**) Sample with 20cN conductive yarn input tension; (**b**) Sample with 10 cN conductive yarn input tension; (**c**) Sample with 5 cN conductive yarn input tension.

As can be seen from Table 6, when the conductive yarn input tension was increased, the electrical resistance of the samples increased. A number of aspects of the work have been considered that may help to elucidate this situation. Firstly, it should be noted that the elastomeric yarn input tension has been kept unaltered, at 8 cN, for all three sample types and only the conductive yarn input tension has been altered. Increasing the conductive yarn input tension enabled the creation of a comparatively short conductive stitch length. Since the conducting loops are located on an interlock base structure, smaller stitches caused a reduction in the conductive contact areas between neighboring knitted loops as may be seen in Figure 5a. Conversely, the insertion of knitted stitches at higher input tensions created stitches that were more uniform and they exhibited “V” shapes with fewer contact points, as is also clearly visible in Figure 6a. At lower input tension levels, the conductive loops adopted a looser arrangement and the legs of the conductive loops provided an increased contact area, as shown in the photomicrographs of Figure 6b,c. According to Holm's contact theory, the reduced contact area results in higher contact resistance and the consequence of that is that knitting with higher conductive yarn input tensions produces knitted sensors with higher electrical resistance.

**Figure 7.**
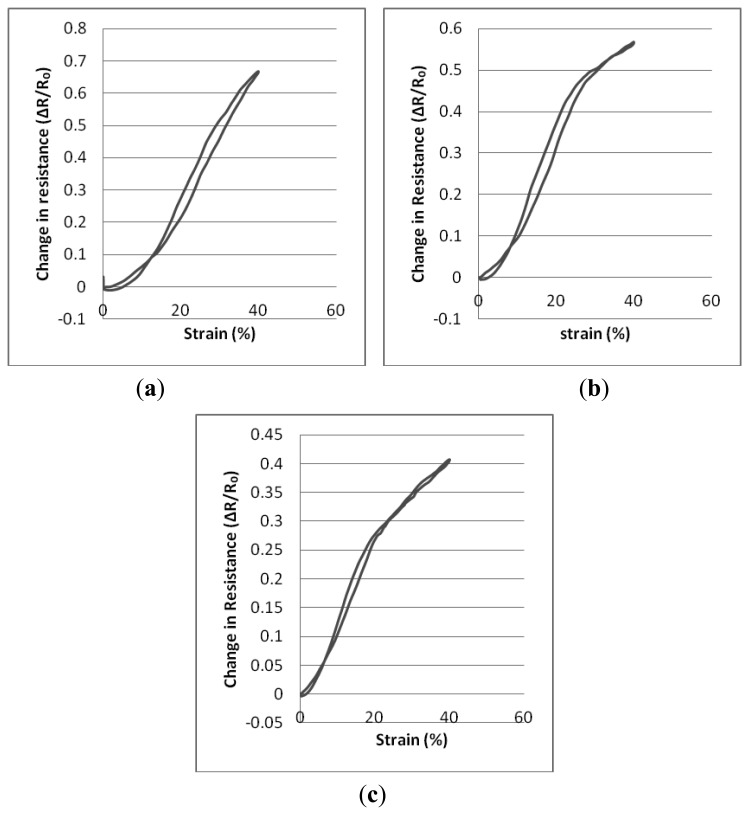
Relative change in resistance-strain graphs of the sensors (**a**) 5 cN conductive yarn input tension; (**b**) 10 cN conductive yarn input tension; (**c**) 20 cN conductive yarn input tension.

Tables 7, 8, and 9 present description of the resistance-strain curves of the sensors in terms of their statistical parameters. Statistical analysis and tests have been performed on sensors which have been created using 800 dtex core Lycra elastomeric yarn to form the interlock base structures. The parameters of the conducting yarn have been set at 5 cN, 10 cN and 20 cN as previously. Hence three variations of the knitted sensor have been created. The graphs in Figure 7 each describe the averaged results of 20 sets of measurements in which the change in resistance is monitored during strain cycling.

In this case, the sensors were characterised by two linear regions within their working range. It may also be observed that the starting points of the second linear region commence at the strain values where the first linear region terminates. Also, maximum hysteresis values of sensors are 3.60%, 2.53% and 1.90% for sensors produced with 5 cN, 10 cN, and 20 cN conductive yarn input tension respectively. Thus, higher conductive yarn input tension enables lower hysteresis values for knitted sensors.

As can be seen from Tables 7, 8, and 9 and from the graphs in Figure 7, the starting value of the working range is higher with samples that have been created with the lowest conductive yarn input tensions. It should be noted that the first linear working range starts from the finishing value of the first hysteresis loop. This behavior derives from the fact that when the input tension of the conductive yarn is increased, this contributes to the production of knitted structures that are more dimensionally stable. Thus, buckling due to the deformation of the sample under cyclic test is reduced compared with those in samples produced with lower levels of conductive yarn input tension. During the unloading stage, the electrical resistance of samples decreases due to the enhanced contact of adjacent conductive loops. However, when the sample starts to buckle, it causes separation of the conductive loops and separation of the knitted conductive loops contributes to an increasing level of electrical resistance. Thus, these opposing mechanisms cause compensation of the electrical resistance to some degree and the change in electrical resistance does not increase to the same extent as when the fabric is being loaded. Hence, when a sample starts to buckle, it creates an electrical hysteresis loop as seen in Figure 7.

Another observation from the graphs in Figure 7 is that the first linear region of the samples reaches higher levels if a lower conductive yarn input tension is applied. This phenomenon occurs as a result of the lower tension which enables the creation of a greater contact area between the conductive loops due to the loose loop structure which increases the contact area between adjacent conductive loops. Since the conductive loops start to separate from their upper parts, *i.e.*, the legs separate from the neighboring legs and the heads separate from adjacent heads. The separation behavior of these parts determines the first linear working range of the sensors. Thus, an enhanced contact between these parts increases the working range of the first linear region and also leads to enhanced gauge factor values. Also, all three types of knitted sensor have higher gauge factor values over their first linear working range than over the extent of their second working range. The reason for this is that after the first linear region, the separation of the sinker loops determines the extent of the change in the electrical resistance. However, the contact area of the sinker loops is smaller than that of the upper parts and separation of these contacts requires higher strain values. Thus, these factors cause relatively lower gauge factor values to be produced.

### 3.3. Effect of Conductive Course Number on Sensing Properties

**Figure 8.**
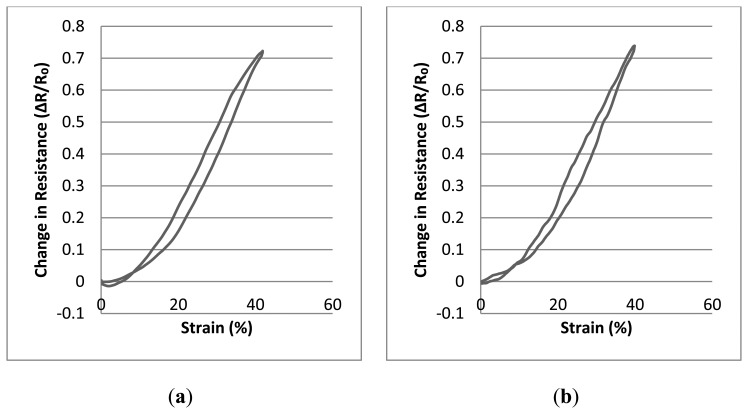
Relative change in resistance-strain graphs of the sensors (**a**) Sensors realised with six conductive courses; (**b**) Sensors realised with four conductive courses.

In this section, the consequence of varying the number of knitted conducting courses in the fabric sensors has been explored. Examples with four and six courses of conducting yarn have been created in order to investigate the effect of this parameter on the sensing properties. Tables 10 and 11 present the statistical results of this investigation. Tests and statistical analysis has been performed as previously. The graphs displayed in Figure 8 describe averaged values of 20 consecutive measurements of change in resistance as cyclic strain testing is performed.

As seen from Tables 10, 11 and from the graphs in Figure 8, the gauge factor values and R**^2^** values are remarkably similar for both types of sensor. However, the starting point of the linear working range is slightly higher for those samples which have been produced with six conductive courses. This situation probably derives from the fact that an increased number of conductive courses affect the tension of the knitted samples as the elastomeric yarn and the conductive yarn contribute to the build-up of internal tension. Hence, increasing the number of conductive courses creates a greater propensity to buckle and this has a measureable effect on the starting point of the linear working range.

## 4. Conclusions

In this paper, different types of textile-based strain sensors have been described. The effects of various production parameters on the sensing properties have been examined for a number of different designs of sensor. These knitted structures demonstrate cyclic properties that offer significant levels of change in resistance and are furthermore largely free of drift; they may hence be suitable for the measurement of human body articulations or physiological signals. Therefore, specific production parameters should be chosen for specific areas of application. Variations in elastomeric yarn type, particularly those made by different manufacturers differ significantly from one another; they affect the sensing properties and alter fundamental parameters such as the gauge factor, linearity and working range. Knitted structures which have lower extension values at a given force value demonstrated lower gauge factor values and reduced working ranges. Samples realised with 800 dtex core spun Lycra yarn produced samples with the highest gauge factor due to the enhanced contact area between the conductive loops. Another important observation is that the input tension applied to a conductive yarn during manufacture of a given design has a considerable effect on the sensing properties. The knitted sensors realised with 5 cN conductive yarn input tension demonstrated the highest gauge factor values. The mechanism underlying this effect is that the loose arrangement of conductive knitted loops increases the contact area between successive loops in the conducting course. However, the starting levels of the first linear working range of such samples were higher than for other types of sensor due to the increased tendency to buckle. The last observation concerns the effect of the number of conductive courses inserted into a given interlock base structure. Although the linearity and the gauge factor values do not change significantly, the starting values of the linear working range are higher for designs with six courses of conductive yarn as the elastomeric base yarn and the conductive yarn have different tensile properties.

## Figures and Tables

**Table 1.** Production parameters of sensing structures.

	**Core lycra Linear Yarn Density (dtex)**	**Elastomeric Yarn Input Tension (cN)**	**Number of Conductive Wales**	**Number of Conductive Courses**
Type 1	800	8	36	6
Type 2	570	8	36	6
Type 3	156	8	36	6

**Table 2.** Elastomeric yarn elongation values at 8 cN applied tension.

**Elastomeric Yarn Type**	**Applied Force (cN)**	**Extension (%)**
800 dtex core Lycra with double PA 6.6 covering	8 cN	175.2
570 dtex core Lycra with double PA 6.6 covering	8 cN	260.97
156 dtex core Lycra with double PA 6.6 covering	8 cN	67.17

**Table 3.** Statistical results from the Type 1 sensor.

**Linear Working Range**	**Working Range Starting Point (%)**	**Gauge Factor Value**	**R^2^ Value**
Average Value	8.405	2.261	0.9948
Standard Error	0.091	0.055	0.0005
95% confidence interval(max)	8.562	2.282	0.9950
95% confidence interval (min)	8.247	2.240	0.9946

**Table 4.** Statistical results of Type 2 sensor.

	**Working Range Starting Point (%)**	**Gauge Factor Value**	**R^2^ Value**
Average Value	2.624	0.864	0.9942
Standard Error	0.097	0.0018	0.0002
95% confidence interval(max)	2.792	0.8671	0.9946
95% confidence interval (min)	2.455	0.8608	0.9938

**Table 5.** Statistical results of Type 3 sensor.

**First Working Range**	**Working Range Starting Point (%)**	**Gauge Factor Value**	**R^2^ Value**
Average Value	3.324	0.234	0.9929
Standard Error	0.122	0.0018	0.0004
95% confidence interval(max)	3.535	0.2371	0.9936
95% confidence interval (min)	3.112	2.2308	0.9922

**Second Working Range**	**Working Range Starting Point (%)**	**Gauge Factor Value**	**R^2^ Value**

Average Value	16.782	0.0804	0.94716
Standard Error	0.2824	0.0001	0.00005
95% confidence interval(max)	17.270	0.0805	0.9472
95% confidence interval (min)	16.293	0.0802	0.9470

**Table 6.** Electrical resistance values of samples prior to extension.

**Conductive Yarn Input Tension (cN)**	**Electrical Resistance Values (ohm)**
5 cN	124.5
10 cN	159.7
20 cN	170.1

**Table 7.** Statistical results of samples with conductive yarn of 5 cN input tension.

**First Working Range**	**Working Range Starting Point (%)**	**Gauge Factor Value**	**R^2^ Value**
Average Value	11.984	2.549	0.9983
Standard Error	0.2039	0.014	0.0001
95% confidence interval(max)	12.336	2.573	0.9984
95% confidence interval (min)	11.631	2.524	0.9982

**Second Working Range**	**Working Range Starting Point (%)**	**Gauge Factor Value**	**R^2^ Value**

Average Value	29.975	1.554	0.9900
Standard Error	0.2296	0.012	0.0027
95% confidence interval(max)	30.205	1.575	0.9911
95% confidence interval (min)	29.745	1.533	0.9890

**Table 8.** Statistical results of samples with conductive yarn of 10 cN input tension.

**First Working Range**	**Working Range Starting Point (%)**	**Gauge Factor Value**	**R^2^ Value**
Average Value	7.9645	2.534	0.9976
Standard Error	0.1863	0.011	0.0002
95% confidence interval(max)	8.2867	2.554	0.9980
95% confidence interval (min)	7.6422	2.514	0.9973

**Second Working Range**	**Working Range Starting Point (%)**	**Gauge Factor Value**	**R^2^ Value**

Average Value	22.071	0.764	0.9783
Standard Error	0.1269	0.009	0.0010
95% confidence interval(max)	22.290	0.780	0.9801
95% confidence interval (min)	21.851	0.748	0.9764

**Table 9.** Statistical results of samples with conductive yarn of 20 cN input tension.

**First Working Range**	**Working Range Starting Point (%)**	**Gauge Factor Value**	**R^2^ Value**
Average Value	6.823	1.863	0.9958
Standard Error	0.123	0.006	0.0002
95% confidence interval(max)	7.036	1.874	0.9962
95% confidence interval (min)	6.609	1.851	0.9953

**Second Working Range**	**Working Range Starting Point (%)**	**Gauge Factor Value**	**R^2^ Value**

Average Value	17.665	0.685	0.9942
Standard Error	0.1121	0.003	0.0001
95% confidence interval(max)	17.859	0.691	0.9945
95% confidence interval (min)	17.471	0.679	0.9939

**Table 10.** Statistical results of samples realised with six conductive courses.

	**Working Range Starting Point (%)**	**Gauge Factor Value**	**R^2^ Value**
Average Value	8.405	2.261	0.9948
Standard Error	0.091	0.055	0.0005
95% confidence interval(max)	8.562	2.282	0.9950
95% confidence interval (min)	8.247	2.240	0.9946

**Table 11.** Statistical results of samples realised with four conductive courses.

	**Working Range Starting Point (%)**	**Gauge Factor Value**	**R^2^ Value**
Average Value	7.084	2.250	0.9953
Standard Error	0.120	0.015	0.0002
95% confidence interval(max)	7.291	2.276	0.9957
95% confidence interval (min)	6.963	2.234	0.9951

## References

[1] Van Langenhove L., Hertleer C. (2004). Smart clothing: A new life. Int. J. Cloth. Sci. Technol..

[2] Hoffmann T., Eilebrecht B., Leonhardt S. (2011). Respiratory Monitoring System on the Basis of Capacitive Textile Force Sensors. IEEE Sens. J..

[3] Witt J., Narbonneau F., Schukar M., Krebber K., De Jonckheere J., Jeanne M., Kinet D., Paquet B., Depre A. (2012). Medical Textiles with Embedded Fiber Optic Sensors for Monitoring of Respiratory Movement. IEEE Sens. J..

[4] Ziegler S., Frydrysiak M. (2009). Initial Research into the Structure and Working Conditions of Textile Thermocouples. Fibres Text. East. Eur..

[5] Sibinski M., Jakubowska M., Sloma M. (2010). Flexible Temperature Sensors on Fibers. Sensors.

[6] Chiarugi F., Karatzanis I., Zacharioudakis G., Meriggi P., Rizzo F., Stratakis M., Louloudakis S., Biniaris C., Valentini M., Di Rienzo M. Measurement of heart rate and respiratory rate using a textile-based wearable device in heart failure patients.

[7] Peltokangas M., Verho J., Vehkaoja A. (2012). Night-Time EKG and HRV Monitoring with Bed Sheet Integrated Textile Electrodes. IEEE Trans. Inf. Technol. Biomed..

[8] Mattmann C., Amft O., Harms H., Troster G., Clemens F. Recognizing upper body postures using textile strain sensors.

[9] Meyer J., Arnrich B., Schumm J., Troster G. (2010). Design and modeling of a textile pressure sensor for sitting posture classification. IEEE Sens. J..

[10] Atalay O., Kennon W., Husain M. (2013). Textile-Based Weft Knitted Strain Sensors: Effect of Fabric Parameters on Sensor Properties. Sensors.

[11] Dias T., Beattie P.C.W., Cooke W., Wijesiriwardana R., Mitcham K., Mukhopadhyay S., Hurley W. (2004). Knitted Transducer Devices.

[12] Pacelli M., Loriga G., Paradiso R. Flat knitted sensors for respiration monitoring.

[13] Yang K., Song G.-L., Zhang L., Li L.-W. Modelling the electrical property of 1× 1 rib knitted fabrics made from conductive yarns.

[14] Zysset C., Cherenack K., Kinkeldei T., Troster G. Weaving integrated circuits into textiles.

[15] Zysset C., Kinkeldei T., Münzenrieder N., Petti L., Salvatore G., Tröster G. (2013). Combining electronics on flexible plastic strips with textiles. Text. Res. J..

[16] Yamashita T., Miyake K., Itoh T. Conductive polymer coated elastomer contact structure for woven electronic textile.

[17] Li L.-F., Ding Y.-S. Design and Analysis of Parallel Woven Structure-Based Flexible Resistive Pressure Sensor.

[18] Cherenack K., Zysset C., Kinkeldei T., Münzenrieder N., Tröster G. (2010). Woven Electronic Fibers with Sensing and Display Functions for Smart Textiles. Adv. Mater..

[19] Kannaian T., Neelaveni R., Thilagavathi G. (2013). Design and development of embroidered textile electrodes for continuous measurement of electrocardiogram signals. J. Ind. Text..

[20] Strazdienė E., Blaževic P., Vegys A., Dapkuniene K. (2007). New tendencies of wearable electronics application in smart clothing. J. Electron. Electr. Eng..

[21] Post E.R., Orth M., Russo P.R., Gershenfeld N. (2000). E-broidery: Design and fabrication of textile-based computing. IBM Syst. J..

[22] Salibindla S., Ripoche B., Lai D.T.H., Maas S. Characterization of a new flexible pressure sensor for body sensor networks.

[23] Alagirusamy R., Eichhoff J., Gries T., Jockenhoevel S. (2013). Coating of conductive yarns for electro-textile applications. J. Text. Inst..

[24] Devaux E., Koncar V., Kim B., Campagne C., Roux C., Rochery M., Saihi D. (2007). Processing and characterization of conductive yarns by coating or bulk treatment for smart textile applications. Trans. Inst. Meas. Control.

[25] Metcalf C.D., Collie S.R., Cranny A.W., Hallett G., James C., Adams J., Chappell P.H., White N.M., Burridge J.H. Fabric-based strain sensors for measuring movement in wearable telemonitoring applications.

[26] Neumann J.J., Greve D.W., Oppenheim I.J. (2004). Comparison of piezoresistive and capacitive ultrasonic transducers. Proc. SPIE.

[27] Gu J.F., Gorgutsa S., Skorobogatiy M. (2011). A fully woven touchpad sensor based on soft capacitor fibers. http://www.arxiv.org/abs/1106.3881.

[28] Sergio M., Manaresi N., Tartagni M., Guerrieri R., Canegallo R. A textile based capacitive pressure sensor.

[29] Note L.P.T. (2004). Capacitive Sensor Operation and Optimization.

[30] Hardy V.-G. (2008). Design and Construction of Smart Structures for Technical Textiles. Ph.D. Thesis.

[31] Hoffmann K. (1989). An Introduction to Measurements Using Strain Guages.

[32] Mattmann C., Clemens F., Tröster G. (2008). Sensor for measuring strain in textile. Sensors.

[33] Melnykowycz M., Koll B., Scharf D., Clemens F. (2014). Comparison of Piezoresistive Monofilament Polymer Sensors. Sensors.

[34] Katragadda R.B., Xu Y. (2008). A novel intelligent textile technology based on silicon flexible skins. Sens. Actuators A Phys..

[35] Schwarza A., Kazania I., Cunyb L., Hertleera C., Ghekiereb F., De Clercqb G., De Meyc G., Van Langenhovea L. (2011). Electro-conductive and elastic hybrid yarns–The effects of stretching, cyclic straining and washing on their electro-conductive properties. Mater. Des..

[36] Guo L., Berglin L., Mattila H. (2012). Improvement of electro-mechanical properties of strain sensors made of elastic-conductive hybrid yarns. Text. Res. J..

[37] Zhang H., Tao X., Yu T., Wang S. (2006). Conductive knitted fabric as large-strain gauge under high temperature. Sens. Actuators A Phys..

[38] Wijesiriwardana R., Dias T., Mukhopadhyay S. Resistive fibre-meshed transducers.

[39] Zhang H., Tao X. From wearable to aware: Intrinsically conductive electrotextiles for human strain/stress sensing.

[40] Holm R. (1967). Electric Contacts: Theory and Applications.

